# An Aggressive Case of Sinonasal Squamous Cell Carcinoma, Invasive to Bone, Arising Within Inverted Papilloma with Intracranial Extension: A Case Report

**DOI:** 10.7759/cureus.4508

**Published:** 2019-04-20

**Authors:** Katherine Garcia de de Jesus, Sorab Gupta, Richard R Hwang, Ivette Vigoda, Oscar Cisneros

**Affiliations:** 1 Internal Medicine, St. Barnabas Hospital Health System / Albert Einstein College of Medicine, Bronx, USA; 2 Oncology, St. Barnabas Hospital Health System / Albert Einstein College of Medicine, Bronx, USA; 3 Pathology, St. Barnabas Hospital Health System / Albert Einstein College of Medicine, Bronx, USA

**Keywords:** sinonasal, squamous, carcinoma, papilloma

## Abstract

Sinonasal squamous cell carcinoma represents a rare and aggressive disease. Clinical presentation usually mimics other benign entities and consequently this malignancy is seldom diagnosed in early stages. Surgical management, although is standard of care, is rarely amenable due to the structures involved, usually intracranially. This article encompasses a case report of squamous cell carcinoma involving the ethmoidal, maxillary, and sphenoid sinuses invasive to bone and extending intracranially.

## Introduction

Squamous cell carcinomas (SCCs) of the sinuses represent a rare and aggressive malignancy. Risk factors associated with SCCs are smoking, human papilloma virus (HPV) infection, and occupational exposure to nickel and welding fumes [1]. The standard of care is surgery, when amenable, followed by radiotherapy [2]. The clinical presentation can be very unspecific, often times it is misdiagnosed with inflammatory sinus disease, which delays treatment. Symptoms reported by patients are epistaxis, pain, swelling, foreign body sensation, and nasal obstruction, among others [1]. This disease is usually diagnosed late, when some vital structures are compromised, making the treatment more challenging. ﻿Sinonasal inverted papilloma is a locally aggressive tumor arising from the Schneiderian membrane that lines the nasal cavity and paranasal sinuses. Endoscopic resection is the gold standard in the treatment of inverted papilloma. ﻿The complexity of the anatomy of the sinonasal region and low incidence of these tumors represents a diagnostic and therapeutic challenge. ﻿This area is also close to the frontal cortex through the cribriform plate of the ethmoid bone and is closely connected ﻿by several vascular and lymphatic structures. The maxillary sinus is the most commonly affected sinus and ethmoid sinus the least involved [2].

## Case presentation

**Figure 1:**
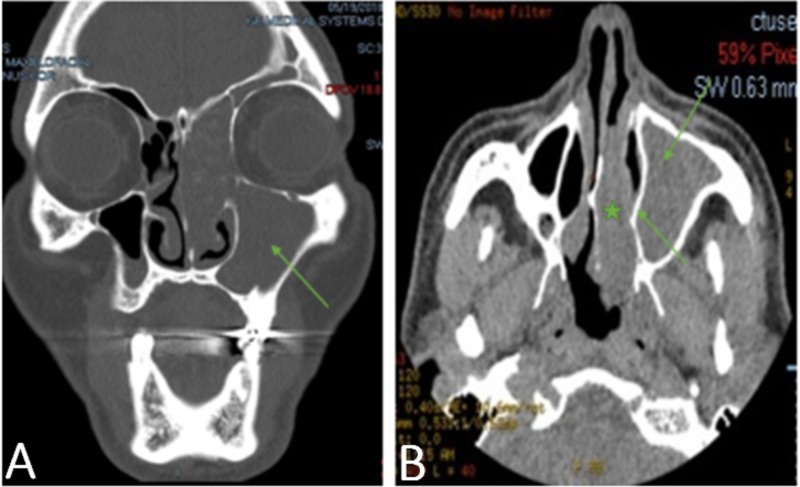
Non-contrast maxillofacial computed tomography (CT) scan Coronal (Figure A) and axial (Figure B) non-contrast maxillofacial CT scan demonstrates near-complete opacification of the left ethmoid and maxillary sinus, suggesting malignancy involving maxillary sinus and the left nasal cavity. Figure B shows septum deviation secondary to mass effect.

**Figure 2:**
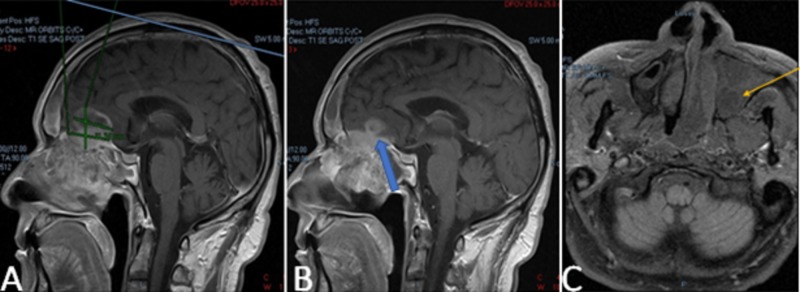
Magnetic resonance imaging (MRI) of the brain and orbits MRI of the brain and orbits demonstrates an aggressive, destructive, heterogeneously predominantly cystic sinonasal malignancy. A. Sagittal MRI revealing ethmoid sinus involvement spreading to the brain. B. Interval intracranial extension through destruction of cribriform plate (blue arrow). C. Midline irregular mass adherent to nasal septum, with septum deviation and surrounding edema (yellow arrow).

We present a 65-year-old man from Morocco with no significant medical history, a non smoker, who presented initially with a complaint of left-sided facial pain and frontotemporal headaches associated with diplopia for one month. Review of his system was positive also for nasal stuffiness, weight loss, and intermittent epistaxis. The patient reported being treated in the past empirically for presumed sinusitis, with no significant relief. On examination, a left eye lateral gaze paralysis was noted; otherwise the rest of the physical exam was reported with no alterations. A maxillofacial computed tomography (CT) scan revealed complete opacification of the left ethmoid, sphenoid, maxillary and frontal sinus with osseous erosion of cribriform plate, as can be seen in Figures 1A-1B. Brain and orbits magnetic resonance imaging (MRI) demonstrated a destructive sinonasal malignancy with extension into the bilateral orbits and showing inferior components seen adherent to either side of the nasal septum with intracranial extension, as can be perceived in Figures 2A-2C. An MRI soft tissue of the neck revealed the mass destroying the hard palate of the maxilla and extending into the bilateral ethmoidal sinuses, maxillary sinuses, anterior walls of the sphenoid sinuses as well as medial walls of the bilateral orbits, deviating the medial recti medially and extending intracranially into the anterior cranial fossa through the cribriform plate.

**Figure 3:**
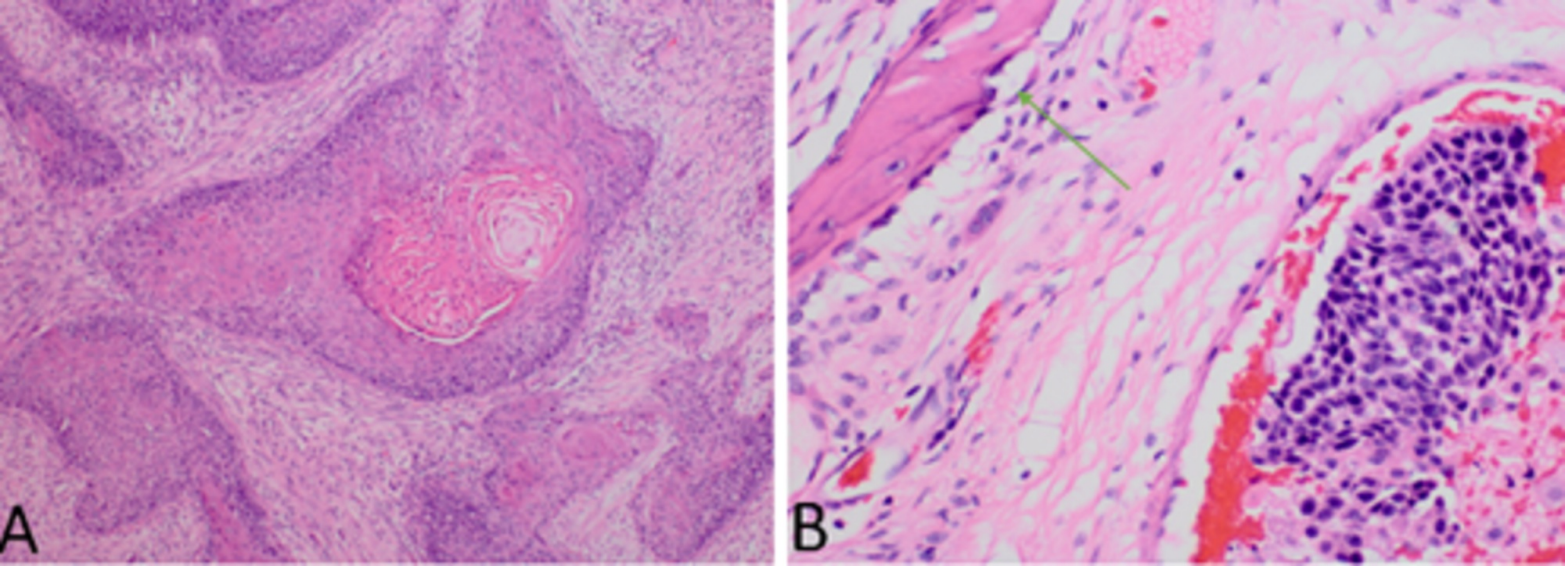
Nasal contents pathology Pathology of nasal contents revealed fragments of inverted papilloma with dysplastic features and foci suspicious for an invasive squamous cell carcinoma. Invasion to bone is noted in Figure B (green arrow).

Positron emission tomography (PET) scan was performed demonstrating bilateral involvement of ethmoidal and sphenoidal sinuses, with intracranial bifrontal parafalcine extension. No signs of lymph node involvement were shown on the PET scan. He underwent Functional Endoscopic Sinus Surgery with debulking of the nasal mass. Pathology was amended as moderately differentiated squamous cell carcinoma, invasive to the bone, arising within sinonasal papilloma showing inverted and exophytic features, as can be seen in Figures 3A-3B. Based on the clinical and histopathological features, the disease was classified as T4bM0, locally advanced sinus SCC. Due to the extension of the disease, surgery was not warranted. The patient was started on induction chemotherapy with docetaxel, cisplatin, and fluorouracil with good tolerance and resulting in resolution of the facial palsy. Repeated images revealed a subsequent interval decrease in the overall size of the tumor, but significant persistent disease with continuous invasion into the adjacent brain parenchyma. Although not enough data to support treatment is available, the strategy now is to continue concurrent chemotherapy and radiotherapy.

## Discussion

Paranasal malignancies represent a rare and aggressive entity, accounting roughly for 3% of all head and neck neoplasias, affecting mostly male patients in the sixth decade of life. The most predominant type is squamous cell carcinoma (SCC). About 10% of SCCs are associated with inverted papilloma. The tumors frequently arise in air filled cavities, most commonly within the maxillary sinus, causing insidious local invasion with late presentation of signs and symptoms. Consequently, most patients are diagnosed with locally advanced disease and frequently disseminated to surrounding structures. Affected areas involve the nasal cavity, maxillary, ethmoid, frontal and sphenoid sinuses, oral cavity, and base of the skull through the cribriform plate. Since the risk of central nervous system involvement is usually imminent, management becomes more challenging [2,3,4].

Initial clinical manifestations are unspecific and occasionally mimic chronic sinusitis. As the disease progresses and invades the surrounding structures, the patient may develop more severe symptoms as exophthalmos, visual disorders as diplopia and respiratory failure. Our patient presented at an advanced stage and was endorsing visual symptoms and nasal obstruction [3].

Risk factors linked with SCC development include prior environmental factors as exposure to wood dust, industrial fumes, textile dust, and asbestos. Chronic human papilloma virus (HPV) infection has also been related to SCC. Some published studies have evaluated the expression of p53 mutation in SCC, but results have been inconclusive with no significant prognostic value. However, p53 was found to be associated with increased risk of failure to therapy and local recurrence [5].

Diagnostic imaging should be advised to determine tumor qualities and for better visualization of the invasion of immediate structures. Computed tomography (CT) and magnetic resonance imaging (MRI) are the tools of choice in such situations. CT is preferred over MRI for its capacity to reveal bone invasion and calcifications [3].

Owing to the rapid, aggressive local spread and tendency for local relapse, the purpose of treatment and the most valuable factor to improve survival is to achieve local control. Paranasal tumors comprise just around 0.5% of all malignancies, and the treatment for paranasal tumors remains controversial. Standard care consists of surgery, radiation therapy, and chemotherapy. If the tumor is positioned in a reachable location, endoscopic surgical intervention can be attempted; still radical surgery continues to be preferred whenever it is possible. In case of intracranial invasion, as in our patient, skull base surgery is limited due to nearby vital structures, labeling them as unresectable. For those patients, radiotherapy alone may play an important role by debulking the tumor; however, the high dose of radiation required may yield brain and optic nerve injury. Intra-arterial chemoradiotherapy (IA-CRT) has demonstrated favorable local control for advanced disease, but no definitive prognostic value has been defined. In more advanced cases, systemic chemotherapy followed by concurrent chemotherapy with radiotherapy is aimed to reduce tumor burden as much as possible, improve quality of life, as well as avert distant metastasis. Due to the rarity of the disease, not enough clinical data are available regarding the benefit of immunotherapy or proton beam therapy and in most cases, recommendations for treatment are mostly extrapolated from other types of head and neck malignancies [6,7,8]. Nevertheless, even after the use of a multimodality approach the prognosis continues to be unfavorable, especially if cervical lymph node metastasis is present. Prophylactic neck irradiation has been proposed in prior clinical studies, but no clear recommendations exist yet [9,10].

## Conclusions

Squamous cell carcinomas (SCCs) of the nasal cavity and sinuses are rare tumors that must be recognized early in the course of the disease in order to improve management outcomes. Physicians should have these conditions in mind and pay close attention to the symptoms because those are nonspecific and can delay proper diagnosis. Careful physical examination and imaging modalities should be made promptly to improve patient clinical outcomes.
